# Bis[4-amino-*N*-(pyrimidin-2-yl)benzene­sulfonamidato](1,10-phenanthroline)nickel(II)

**DOI:** 10.1107/S1600536812008185

**Published:** 2012-02-29

**Authors:** Xiu-Feng Hu, Yue Bing, Xing Li, Zu-Ping Kong

**Affiliations:** a Faculty of Materials Science and Chemical Engineering, Ningbo University, Ningbo, Zhejiang 315211, People’s Republic of China

## Abstract

In the mononuclear title compound, [Ni(C_10_H_9_N_4_O_2_S)_2_(C_12_H_8_N_2_)], the Ni^II^ atom has a distorted octa­hedral coordination geometry comprising four N atoms from two 4-amino-*N*-(pyrimidin-2-yl)benzene­sulfonamidate ligands and two N atoms from a 1,10-phenanthroline ligand. In the crystal, mol­ecules are connected into a three-dimensional supra­molecular network *via* N—H⋯O hydrogen bonds and weak C—H⋯O and C—H⋯N contacts.

## Related literature

For related literature regarding the properties of 4-amino-*N*-(pyrimidin-2-yl)benzene­sulfonamidate ligands, see: Ellena *et al.* (2007 ▶); Garcia-Raso *et al.* (1997 ▶). For related literature regarding crystal engineering studies of 4-amino-*N*-(pyrimidin-2-yl) benzene­sulfonamidate ligands, see: Garcia-Raso *et al.* (2000 ▶); Golzar Hossain *et al.* (2007 ▶); Gutierrez *et al.* (2001 ▶).
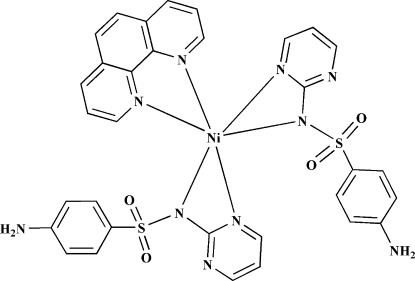


## Experimental

### 

#### Crystal data

[Ni(C_10_H_9_N_4_O_2_S)_2_(C_12_H_8_N_2_)]
*M* *_r_* = 737.46
Orthorhombic, 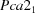

*a* = 11.015 (2) Å
*b* = 17.995 (3) Å
*c* = 16.128 (3) Å
*V* = 3196.9 (10) Å^3^
*Z* = 4
Mo *K*α radiation
μ = 0.79 mm^−1^
*T* = 293 K
0.34 × 0.26 × 0.18 mm

#### Data collection

Bruker SMART APEXII CCD area-detector diffractometer
Absorption correction: multi-scan (*ABSCOR*; Higashi, 1995 ▶) *T*_min_ = 0.774, *T*_max_ = 0.870
27217 measured reflections
7075 independent reflections
5020 reflections with *I* > 2σ(*I*)
*R*_int_ = 0.058

#### Refinement

*R*[*F* ^2^ > 2σ(*F* ^2^)] = 0.044
*wR*(*F* ^2^) = 0.110
*S* = 1.02
7075 reflections
431 parameters
2 restraints
H-atom parameters constrained
Δρ_max_ = 0.51 e Å^−3^
Δρ_min_ = −0.35 e Å^−3^
Absolute structure: Flack (1983 ▶), 3236 Friedel pairs
Flack parameter: 0.236 (16)

### 

Data collection: *SMART* (Bruker, 2001 ▶); cell refinement: *SAINT* (Bruker, 2001 ▶); data reduction: *SAINT*; program(s) used to solve structure: *SHELXS97* (Sheldrick, 2008 ▶); program(s) used to refine structure: *SHELXL97* (Sheldrick, 2008 ▶); molecular graphics: *SHELXTL* (Sheldrick, 2008 ▶); software used to prepare material for publication: *SHELXTL*.

## Supplementary Material

Crystal structure: contains datablock(s) I, global. DOI: 10.1107/S1600536812008185/ez2279sup1.cif

Structure factors: contains datablock(s) I. DOI: 10.1107/S1600536812008185/ez2279Isup2.hkl

Additional supplementary materials:  crystallographic information; 3D view; checkCIF report

## Figures and Tables

**Table 1:** Hydrogen-bond geometry (Å, °)

*D*—H⋯*A*	*D*—H	H⋯*A*	*D*⋯*A*	*D*—H⋯*A*
N1—H1*A*⋯O1^i^	0.86	2.46	3.162 (6)	140
N1—H1*B*⋯O2^ii^	0.86	2.23	3.056 (6)	162
N5—H5*A*⋯O3^iii^	0.86	2.52	3.285 (6)	148
N5—H5*B*⋯O4^iv^	0.86	2.20	3.016 (6)	158
C5—H5*C*⋯O2^v^	0.93	2.56	3.377 (5)	147
C12—H12*A*⋯N8^iii^	0.93	2.56	3.470 (6)	166

Symmetry codes: (i) 
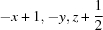
; (ii) 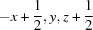; (iii) 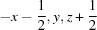; (iv) 
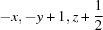
; (v) 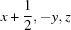.

**Fig. 1.**
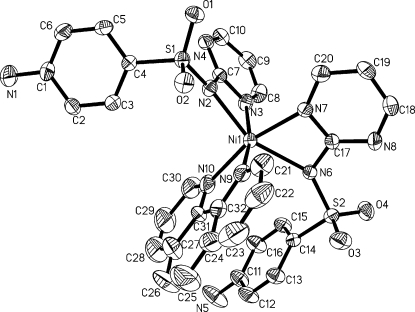
The molecular structure of (I) showing displacement ellipsoids at the 30% probability level.

**Fig. 2.**
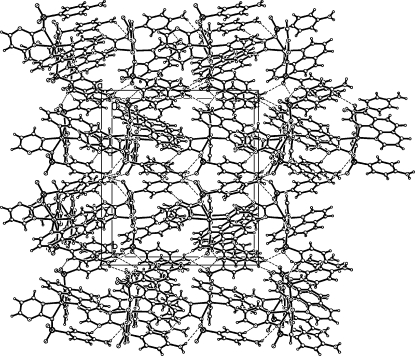
Unit-cell contents for (I) viewed in projection down the a axis.

## supplementary crystallographic information

### Comment

4-amino-*N*-(pyrimidin-2-yl)benzenesulfonamidato ligands have strong metal-binding properties as well as antibacterial functions (Ellena *et al.*, 2007; Garcia-Raso *et al.*, 1997). Crystal engineering studies of metal 4-amino-*N*-(pyrimidin-2-yl)benzenesulfonamidato complexes are less well developed (Garcia-Raso *et al.*, 2000; Golzar *et al.*, 2007; Gutierrez *et al.* 2001), and adding 1,10-phenanthroline as secondary ligand with 4-amino-*N*-(pyrimidin-2-yl)benzenesulfonamide to synthesize novel complex has not been reported yet, as far as we know. Here, we report the crystal structure of the title nickel complex with 1,10-phenanthroline and 4-amino-*N*-(pyrimidin-2-yl)benzenesulfonamide, (I).

Complex (I) is a mononuclear structure, in which the Ni^II^ atom has a distorted octahedral coordination geometry comprising four N atoms from two 4-amino-*N*-(pyrimidin-2-yl)benzenesulfonamidato ligands, two N atoms from a 1,10-phenanthroline ligand. Ni—N distances range from 2.064 to 2.205 Å (Figure 1). In the crystal, the molecules are connected into a three-dimensional supramolecular network *via* N—H···O hydrogen bonds and weak C—H···O and C—H···N contacts. (Figure 2).

### Experimental

A mixture containing 0.005 mmol of 4-amino-*N*-(pyrimidin-2-yl)benzenesulfonamide, 0.005 mmol of Ni(OAc)_2_, 0.005 mmol of 1,10-phenanthroline and 0.005 mmol of KOH was placed in a small vial containing H_2_O (1.0 ml). The vial was sealed, heated at 373 K for 2 days, and allowed to cool to room temperature. Green crystals suitable for X-ray diffraction were collected and dried in air (yield, 31%).

### Refinement

H atoms attached to C and N atoms were placed in calculated positions and treated using a riding-model approximation [C—H, N—H = 0.93–0.98 Å with *U*_iso_(H) = 1.2U_eq_(C,N)/1.5 *U*_eq_(C)]. The crystal studied was a racemic twin, as suggested by the Flack parameter of 0.236 (16) obtained by the TWIN/BASF procedure in SHELXL (Sheldrick, 2008).

### Crystal data

**Table:**

[Ni(C_10_H_9_N_4_O_2_S)_2_(C_12_H_8_N_2_)]	*F*(000) = 1520
*M**_r_* = 737.46	*D*_x_ = 1.532 Mg m^−^^3^
Orthorhombic, *P**c**a*2_1_	Mo *K*α radiation, λ = 0.71073 Å
Hall symbol: P 2c -2ac	Cell parameters from 4514 reflections
*a* = 11.015 (2) Å	θ = 2.2–23.4°
*b* = 17.995 (3) Å	µ = 0.79 mm^−^^1^
*c* = 16.128 (3) Å	*T* = 293 K
*V* = 3196.9 (10) Å^3^	Block, green
*Z* = 4	0.34 × 0.26 × 0.18 mm

### Data collection

**Table:**

Bruker SMART APEXII CCD area-detector diffractometer	7075 independent reflections
Radiation source: fine-focus sealed tube	5020 reflections with *I* > 2σ(*I*)
Graphite monochromator	*R*_int_ = 0.058
phi and ω scans	θ_max_ = 27.6°, θ_min_ = 2.2°
Absorption correction: multi-scan (*ABSCOR*; Higashi, 1995)	*h* = −14→14
*T*_min_ = 0.774, *T*_max_ = 0.870	*k* = −23→22
27217 measured reflections	*l* = −18→20

### Refinement

**Table:**

Refinement on *F*^2^	Secondary atom site location: difference Fourier map
Least-squares matrix: full	Hydrogen site location: inferred from neighbouring sites
*R*[*F*^2^ > 2σ(*F*^2^)] = 0.044	H-atom parameters constrained
*wR*(*F*^2^) = 0.110	*w* = 1/[σ^2^(*F*_o_^2^) + (0.0454*P*)^2^ + 0.7787*P*] where *P* = (*F*_o_^2^ + 2*F*_c_^2^)/3
*S* = 1.02	(Δ/σ)_max_ = 0.001
7075 reflections	Δρ_max_ = 0.51 e Å^−^^3^
431 parameters	Δρ_min_ = −0.35 e Å^−^^3^
2 restraints	Absolute structure: Flack (1983), 3236 Friedel pairs
Primary atom site location: structure-invariant direct methods	Flack parameter: 0.236 (16)

### Special details

**Table:**

Geometry. All e.s.d.'s (except the e.s.d. in the dihedral angle between two l.s. planes) are estimated using the full covariance matrix. The cell e.s.d.'s are taken into account individually in the estimation of e.s.d.'s in distances, angles and torsion angles; correlations between e.s.d.'s in cell parameters are only used when they are defined by crystal symmetry. An approximate (isotropic) treatment of cell e.s.d.'s is used for estimating e.s.d.'s involving l.s. planes.
Refinement. Refinement of *F*^2^ against ALL reflections. The weighted *R*-factor *wR* and goodness of fit *S* are based on *F*^2^, conventional *R*-factors *R* are based on *F*, with *F* set to zero for negative *F*^2^. The threshold expression of *F*^2^ > σ(*F*^2^) is used only for calculating *R*-factors(gt) *etc*. and is not relevant to the choice of reflections for refinement. *R*-factors based on *F*^2^ are statistically about twice as large as those based on *F*, and *R*- factors based on ALL data will be even larger.

### Fractional atomic coordinates and isotropic or equivalent isotropic displacement parameters (Å^2^)

**Table:**

	*x*	*y*	*z*	*U*_iso_*/*U*_eq_	
Ni1	0.08935 (4)	0.25754 (2)	0.18766 (4)	0.04064 (12)	
S1	0.26208 (7)	0.09491 (5)	0.18621 (8)	0.0421 (2)	
S2	−0.12704 (9)	0.40536 (6)	0.14560 (7)	0.0451 (3)	
O1	0.3501 (3)	0.07865 (16)	0.12315 (19)	0.0571 (8)	
O2	0.1443 (2)	0.06087 (14)	0.1758 (2)	0.0553 (8)	
O3	−0.2414 (2)	0.3700 (2)	0.1294 (2)	0.0745 (10)	
O4	−0.1087 (3)	0.47423 (18)	0.1020 (2)	0.0692 (9)	
N1	0.4429 (4)	−0.0080 (3)	0.5131 (3)	0.0859 (14)	
H1B	0.4044	0.0048	0.5571	0.103*	
H1A	0.5071	−0.0351	0.5168	0.103*	
N2	0.2338 (2)	0.18145 (15)	0.1963 (3)	0.0420 (7)	
N3	0.2674 (3)	0.30325 (16)	0.1940 (3)	0.0466 (7)	
N4	0.4420 (3)	0.22257 (17)	0.1955 (3)	0.0468 (7)	
N5	−0.1094 (5)	0.4790 (3)	0.5019 (3)	0.106 (2)	
H5A	−0.1683	0.4659	0.5337	0.127*	
H5B	−0.0489	0.5033	0.5219	0.127*	
N6	−0.0154 (3)	0.34928 (16)	0.13242 (19)	0.0408 (7)	
N7	0.0896 (3)	0.2665 (2)	0.0604 (2)	0.0460 (9)	
N8	−0.0354 (3)	0.3499 (2)	−0.0161 (2)	0.0583 (10)	
N9	−0.0642 (3)	0.19039 (18)	0.1896 (4)	0.0570 (8)	
N10	0.0438 (5)	0.2643 (2)	0.3117 (3)	0.0667 (13)	
C1	0.4020 (4)	0.0146 (3)	0.4366 (3)	0.0551 (11)	
C2	0.2985 (4)	0.0583 (2)	0.4303 (3)	0.0533 (10)	
H2A	0.2549	0.0705	0.4778	0.064*	
C3	0.2601 (4)	0.0837 (2)	0.3543 (3)	0.0488 (10)	
H3A	0.1919	0.1141	0.3515	0.059*	
C4	0.3206 (3)	0.0651 (2)	0.2817 (3)	0.0395 (9)	
C5	0.4211 (4)	0.0207 (2)	0.2871 (3)	0.0534 (11)	
H5C	0.4623	0.0072	0.2390	0.064*	
C6	0.4622 (4)	−0.0042 (3)	0.3629 (3)	0.0661 (13)	
H6A	0.5310	−0.0340	0.3652	0.079*	
C7	0.3218 (3)	0.23493 (18)	0.1954 (3)	0.0394 (8)	
C8	0.3396 (4)	0.3626 (2)	0.1879 (4)	0.0585 (10)	
H8A	0.3056	0.4099	0.1855	0.070*	
C9	0.4641 (4)	0.3546 (2)	0.1849 (4)	0.0581 (10)	
H9A	0.5152	0.3954	0.1791	0.070*	
C10	0.5090 (3)	0.2838 (2)	0.1909 (3)	0.0522 (9)	
H10A	0.5929	0.2782	0.1919	0.063*	
C11	−0.1119 (4)	0.4612 (3)	0.4200 (3)	0.0624 (13)	
C12	−0.2097 (4)	0.4218 (3)	0.3869 (3)	0.0641 (13)	
H12A	−0.2735	0.4074	0.4211	0.077*	
C13	−0.2117 (4)	0.4043 (2)	0.3044 (3)	0.0540 (11)	
H13A	−0.2776	0.3782	0.2831	0.065*	
C14	−0.1180 (4)	0.4247 (2)	0.2518 (3)	0.0436 (10)	
C15	−0.0206 (4)	0.4629 (2)	0.2838 (3)	0.0524 (11)	
H15A	0.0431	0.4768	0.2492	0.063*	
C16	−0.0166 (4)	0.4810 (3)	0.3674 (3)	0.0657 (13)	
H16A	0.0501	0.5064	0.3886	0.079*	
C17	0.0102 (3)	0.3235 (2)	0.0552 (2)	0.0416 (9)	
C18	0.0034 (5)	0.3153 (3)	−0.0834 (3)	0.0739 (15)	
H18A	−0.0274	0.3313	−0.1340	0.089*	
C19	0.0845 (5)	0.2583 (3)	−0.0851 (3)	0.0763 (17)	
H19A	0.1093	0.2366	−0.1346	0.092*	
C20	0.1284 (5)	0.2342 (3)	−0.0089 (3)	0.0643 (13)	
H20A	0.1845	0.1956	−0.0063	0.077*	
C21	−0.1147 (4)	0.1532 (3)	0.1260 (4)	0.0726 (15)	
H21A	−0.0726	0.1510	0.0761	0.087*	
C22	−0.2264 (5)	0.1177 (3)	0.1312 (6)	0.102 (2)	
H22A	−0.2596	0.0926	0.0862	0.123*	
C23	−0.2840 (6)	0.1216 (4)	0.2044 (7)	0.123 (3)	
H23A	−0.3606	0.1001	0.2086	0.148*	
C24	−0.2353 (8)	0.1563 (5)	0.2753 (6)	0.1249 (18)	
C25	−0.2921 (7)	0.1584 (4)	0.3481 (6)	0.1249 (18)	
H25A	−0.3678	0.1362	0.3543	0.150*	
C26	−0.2388 (8)	0.1930 (4)	0.4128 (6)	0.1249 (18)	
H26A	−0.2788	0.1939	0.4636	0.150*	
C27	−0.1208 (8)	0.2290 (5)	0.4057 (6)	0.116 (2)	
C28	−0.0600 (11)	0.2649 (7)	0.4671 (5)	0.152 (4)	
H28A	−0.0939	0.2655	0.5199	0.182*	
C29	0.0518 (10)	0.3012 (6)	0.4548 (5)	0.130 (3)	
H29A	0.0920	0.3252	0.4979	0.156*	
C30	0.1011 (6)	0.2993 (4)	0.3709 (4)	0.099 (2)	
H30A	0.1741	0.3233	0.3594	0.119*	
C31	−0.0656 (5)	0.2289 (3)	0.3275 (3)	0.0754 (17)	
C32	−0.1222 (5)	0.1917 (3)	0.2603 (4)	0.0717 (15)	

### Atomic displacement parameters (Å^2^)

**Table:**

	*U*^11^	*U*^22^	*U*^33^	*U*^12^	*U*^13^	*U*^23^
Ni1	0.0409 (2)	0.0465 (3)	0.0345 (2)	0.00456 (19)	−0.0015 (3)	0.0027 (3)
S1	0.0427 (4)	0.0406 (5)	0.0428 (5)	−0.0028 (3)	−0.0011 (5)	0.0020 (6)
S2	0.0396 (5)	0.0521 (6)	0.0437 (6)	0.0067 (4)	−0.0002 (4)	−0.0002 (5)
O1	0.0654 (18)	0.0592 (19)	0.0469 (19)	0.0048 (15)	0.0074 (15)	−0.0075 (15)
O2	0.0510 (14)	0.0504 (15)	0.065 (2)	−0.0131 (12)	−0.0083 (16)	−0.0006 (16)
O3	0.0397 (15)	0.125 (3)	0.059 (2)	−0.0106 (17)	−0.0056 (15)	−0.015 (2)
O4	0.102 (3)	0.0514 (19)	0.054 (2)	0.0244 (17)	0.0111 (17)	0.0098 (16)
N1	0.092 (3)	0.112 (4)	0.054 (3)	0.032 (3)	−0.011 (2)	0.016 (3)
N2	0.0408 (14)	0.0349 (15)	0.050 (2)	−0.0026 (11)	−0.0030 (18)	0.0058 (18)
N3	0.0485 (15)	0.0398 (16)	0.051 (2)	−0.0030 (13)	−0.008 (2)	0.003 (2)
N4	0.0402 (14)	0.0541 (18)	0.046 (2)	−0.0020 (13)	−0.0013 (19)	0.002 (2)
N5	0.109 (4)	0.156 (5)	0.052 (3)	−0.066 (4)	0.018 (3)	−0.027 (3)
N6	0.0463 (16)	0.0415 (17)	0.0346 (17)	0.0082 (14)	0.0002 (14)	−0.0013 (15)
N7	0.050 (2)	0.052 (2)	0.036 (2)	0.0120 (16)	0.0017 (16)	−0.0046 (17)
N8	0.069 (2)	0.068 (3)	0.037 (2)	0.019 (2)	−0.0056 (18)	0.0053 (18)
N9	0.0466 (16)	0.0558 (19)	0.068 (2)	0.0068 (14)	0.007 (3)	0.020 (3)
N10	0.083 (3)	0.076 (3)	0.041 (2)	0.030 (2)	−0.007 (2)	0.000 (2)
C1	0.061 (3)	0.050 (3)	0.054 (3)	0.006 (2)	−0.011 (2)	0.009 (2)
C2	0.065 (3)	0.050 (2)	0.045 (3)	0.002 (2)	0.008 (2)	0.003 (2)
C3	0.047 (2)	0.046 (2)	0.053 (3)	0.0064 (19)	0.0030 (19)	0.005 (2)
C4	0.0402 (19)	0.036 (2)	0.042 (2)	0.0011 (16)	−0.0010 (17)	−0.0020 (17)
C5	0.048 (2)	0.057 (3)	0.055 (3)	0.012 (2)	0.007 (2)	−0.003 (2)
C6	0.055 (3)	0.079 (3)	0.064 (3)	0.027 (3)	−0.005 (2)	0.004 (3)
C7	0.0430 (16)	0.0415 (19)	0.034 (2)	−0.0025 (14)	0.001 (2)	−0.001 (2)
C8	0.069 (2)	0.0370 (19)	0.070 (3)	−0.0032 (18)	−0.006 (3)	−0.001 (3)
C9	0.063 (2)	0.051 (2)	0.060 (3)	−0.0197 (18)	0.002 (3)	−0.004 (3)
C10	0.0466 (19)	0.063 (2)	0.048 (2)	−0.0135 (17)	−0.004 (3)	0.000 (3)
C11	0.070 (3)	0.072 (3)	0.045 (3)	−0.020 (2)	0.006 (2)	−0.009 (2)
C12	0.051 (2)	0.087 (4)	0.054 (3)	−0.027 (2)	0.011 (2)	−0.006 (3)
C13	0.045 (2)	0.062 (3)	0.055 (3)	−0.013 (2)	0.003 (2)	−0.007 (2)
C14	0.036 (2)	0.050 (3)	0.045 (2)	0.0035 (18)	0.0027 (17)	0.004 (2)
C15	0.047 (2)	0.057 (3)	0.053 (3)	−0.009 (2)	0.010 (2)	−0.002 (2)
C16	0.058 (3)	0.080 (4)	0.060 (3)	−0.029 (2)	0.007 (2)	−0.008 (3)
C17	0.045 (2)	0.041 (2)	0.038 (2)	0.0024 (16)	0.0008 (17)	0.0032 (18)
C18	0.074 (3)	0.110 (4)	0.037 (3)	0.023 (3)	−0.008 (2)	0.006 (3)
C19	0.086 (4)	0.110 (5)	0.033 (3)	0.027 (3)	0.005 (2)	−0.013 (3)
C20	0.065 (3)	0.076 (3)	0.052 (3)	0.021 (3)	0.002 (2)	−0.009 (3)
C21	0.057 (3)	0.062 (3)	0.100 (5)	−0.010 (2)	−0.010 (3)	−0.002 (3)
C22	0.064 (4)	0.076 (4)	0.168 (8)	−0.005 (3)	−0.011 (4)	0.013 (5)
C23	0.073 (4)	0.072 (4)	0.225 (11)	0.009 (3)	0.041 (6)	0.058 (6)
C24	0.113 (4)	0.126 (4)	0.136 (5)	−0.001 (3)	0.052 (3)	0.040 (4)
C25	0.113 (4)	0.126 (4)	0.136 (5)	−0.001 (3)	0.052 (3)	0.040 (4)
C26	0.113 (4)	0.126 (4)	0.136 (5)	−0.001 (3)	0.052 (3)	0.040 (4)
C27	0.127 (5)	0.134 (6)	0.087 (5)	0.030 (5)	0.030 (5)	0.037 (5)
C28	0.181 (10)	0.225 (12)	0.051 (5)	0.067 (9)	0.008 (6)	−0.001 (6)
C29	0.169 (9)	0.170 (9)	0.052 (5)	0.045 (8)	−0.022 (5)	−0.009 (5)
C30	0.117 (5)	0.130 (6)	0.051 (4)	0.047 (4)	−0.022 (3)	−0.020 (4)
C31	0.083 (4)	0.089 (4)	0.054 (3)	0.037 (3)	0.034 (3)	0.032 (3)
C32	0.069 (3)	0.066 (3)	0.080 (4)	0.020 (3)	0.017 (3)	0.032 (3)

### Geometric parameters (Å, º)

**Table:**

Ni1—N7	2.059 (4)	C5—H5C	0.9300
Ni1—N10	2.066 (4)	C6—H6A	0.9300
Ni1—N9	2.079 (3)	C8—C9	1.379 (5)
Ni1—N2	2.104 (3)	C8—H8A	0.9300
Ni1—N3	2.130 (3)	C9—C10	1.370 (5)
Ni1—N6	2.202 (3)	C9—H9A	0.9300
S1—O1	1.435 (3)	C10—H10A	0.9300
S1—O2	1.444 (2)	C11—C12	1.395 (6)
S1—N2	1.596 (3)	C11—C16	1.396 (6)
S1—C4	1.754 (4)	C12—C13	1.368 (6)
S2—O3	1.435 (3)	C12—H12A	0.9300
S2—O4	1.439 (3)	C13—C14	1.385 (5)
S2—N6	1.605 (3)	C13—H13A	0.9300
S2—C14	1.750 (4)	C14—C15	1.376 (6)
N1—C1	1.374 (6)	C15—C16	1.388 (6)
N1—H1B	0.8600	C15—H15A	0.9300
N1—H1A	0.8600	C16—H16A	0.9300
N2—C7	1.367 (4)	C18—C19	1.360 (7)
N3—C8	1.336 (4)	C18—H18A	0.9300
N3—C7	1.368 (4)	C19—C20	1.391 (7)
N4—C10	1.329 (4)	C19—H19A	0.9300
N4—C7	1.342 (4)	C20—H20A	0.9300
N5—C11	1.360 (6)	C21—C22	1.388 (7)
N5—H5A	0.8600	C21—H21A	0.9300
N5—H5B	0.8600	C22—C23	1.344 (11)
N6—C17	1.359 (5)	C22—H22A	0.9300
N7—C20	1.330 (6)	C23—C24	1.409 (12)
N7—C17	1.349 (5)	C23—H23A	0.9300
N8—C18	1.321 (6)	C24—C25	1.330 (11)
N8—C17	1.343 (5)	C24—C32	1.421 (10)
N9—C32	1.307 (7)	C25—C26	1.350 (11)
N9—C21	1.346 (7)	C25—H25A	0.9300
N10—C30	1.306 (7)	C26—C27	1.457 (12)
N10—C31	1.387 (7)	C26—H26A	0.9300
C1—C2	1.389 (6)	C27—C28	1.359 (13)
C1—C6	1.402 (6)	C27—C31	1.400 (9)
C2—C3	1.374 (6)	C28—C29	1.408 (13)
C2—H2A	0.9300	C28—H28A	0.9300
C3—C4	1.389 (6)	C29—C30	1.459 (10)
C3—H3A	0.9300	C29—H29A	0.9300
C4—C5	1.368 (5)	C30—H30A	0.9300
C5—C6	1.379 (6)	C31—C32	1.418 (8)
			
N7—Ni1—N10	163.95 (16)	N3—C8—H8A	119.7
N7—Ni1—N9	93.55 (19)	C9—C8—H8A	119.7
N10—Ni1—N9	79.7 (2)	C10—C9—C8	117.0 (3)
N7—Ni1—N2	96.67 (15)	C10—C9—H9A	121.5
N10—Ni1—N2	99.08 (17)	C8—C9—H9A	121.5
N9—Ni1—N2	103.64 (11)	N4—C10—C9	125.1 (3)
N7—Ni1—N3	90.95 (16)	N4—C10—H10A	117.5
N10—Ni1—N3	98.85 (19)	C9—C10—H10A	117.5
N9—Ni1—N3	166.70 (12)	N5—C11—C12	120.4 (4)
N2—Ni1—N3	63.36 (11)	N5—C11—C16	121.0 (4)
N7—Ni1—N6	62.52 (12)	C12—C11—C16	118.6 (4)
N10—Ni1—N6	102.74 (14)	C13—C12—C11	120.1 (4)
N9—Ni1—N6	90.91 (12)	C13—C12—H12A	119.9
N2—Ni1—N6	155.62 (13)	C11—C12—H12A	119.9
N3—Ni1—N6	102.27 (12)	C12—C13—C14	121.5 (4)
O1—S1—O2	115.96 (19)	C12—C13—H13A	119.2
O1—S1—N2	113.76 (19)	C14—C13—H13A	119.2
O2—S1—N2	104.49 (15)	C15—C14—C13	118.9 (4)
O1—S1—C4	108.17 (18)	C15—C14—S2	120.7 (3)
O2—S1—C4	107.61 (18)	C13—C14—S2	120.3 (3)
N2—S1—C4	106.3 (2)	C14—C15—C16	120.4 (4)
O3—S2—O4	114.6 (2)	C14—C15—H15A	119.8
O3—S2—N6	111.69 (19)	C16—C15—H15A	119.8
O4—S2—N6	111.68 (18)	C15—C16—C11	120.5 (4)
O3—S2—C14	108.4 (2)	C15—C16—H16A	119.8
O4—S2—C14	107.4 (2)	C11—C16—H16A	119.8
N6—S2—C14	102.18 (19)	N8—C17—N7	124.3 (4)
C1—N1—H1B	120.0	N8—C17—N6	125.9 (3)
C1—N1—H1A	120.0	N7—C17—N6	109.7 (3)
H1B—N1—H1A	120.0	N8—C18—C19	125.8 (5)
C7—N2—S1	123.2 (2)	N8—C18—H18A	117.1
C7—N2—Ni1	94.5 (2)	C19—C18—H18A	117.1
S1—N2—Ni1	140.89 (17)	C18—C19—C20	116.5 (5)
C8—N3—C7	117.4 (3)	C18—C19—H19A	121.8
C8—N3—Ni1	148.6 (3)	C20—C19—H19A	121.8
C7—N3—Ni1	93.3 (2)	N7—C20—C19	119.6 (4)
C10—N4—C7	114.2 (3)	N7—C20—H20A	120.2
C11—N5—H5A	120.0	C19—C20—H20A	120.2
C11—N5—H5B	120.0	N9—C21—C22	123.3 (6)
H5A—N5—H5B	120.0	N9—C21—H21A	118.3
C17—N6—S2	119.7 (3)	C22—C21—H21A	118.3
C17—N6—Ni1	90.3 (2)	C23—C22—C21	116.6 (8)
S2—N6—Ni1	145.00 (19)	C23—C22—H22A	121.7
C20—N7—C17	119.2 (4)	C21—C22—H22A	121.7
C20—N7—Ni1	143.5 (3)	C22—C23—C24	123.9 (7)
C17—N7—Ni1	96.9 (3)	C22—C23—H23A	118.1
C18—N8—C17	114.5 (4)	C24—C23—H23A	118.1
C32—N9—C21	118.2 (4)	C25—C24—C23	123.3 (9)
C32—N9—Ni1	113.6 (4)	C25—C24—C32	123.4 (10)
C21—N9—Ni1	127.9 (4)	C23—C24—C32	113.2 (7)
C30—N10—C31	120.5 (6)	C24—C25—C26	119.4 (9)
C30—N10—Ni1	128.2 (5)	C24—C25—H25A	120.3
C31—N10—Ni1	111.2 (4)	C26—C25—H25A	120.3
N1—C1—C2	120.2 (4)	C25—C26—C27	122.1 (8)
N1—C1—C6	122.3 (4)	C25—C26—H26A	118.9
C2—C1—C6	117.5 (4)	C27—C26—H26A	118.9
C3—C2—C1	120.5 (4)	C28—C27—C31	116.3 (8)
C3—C2—H2A	119.8	C28—C27—C26	126.4 (9)
C1—C2—H2A	119.8	C31—C27—C26	117.3 (9)
C2—C3—C4	121.5 (4)	C27—C28—C29	123.3 (8)
C2—C3—H3A	119.2	C27—C28—H28A	118.3
C4—C3—H3A	119.2	C29—C28—H28A	118.3
C5—C4—C3	118.5 (4)	C28—C29—C30	116.4 (8)
C5—C4—S1	122.1 (3)	C28—C29—H29A	121.8
C3—C4—S1	119.3 (3)	C30—C29—H29A	121.8
C4—C5—C6	120.7 (4)	N10—C30—C29	120.6 (8)
C4—C5—H5C	119.6	N10—C30—H30A	119.7
C6—C5—H5C	119.6	C29—C30—H30A	119.7
C5—C6—C1	121.3 (4)	N10—C31—C27	122.8 (7)
C5—C6—H6A	119.4	N10—C31—C32	117.3 (4)
C1—C6—H6A	119.4	C27—C31—C32	119.9 (7)
N4—C7—N2	125.7 (3)	N9—C32—C24	124.7 (7)
N4—C7—N3	125.5 (3)	N9—C32—C31	117.4 (5)
N2—C7—N3	108.8 (3)	C24—C32—C31	117.8 (7)
N3—C8—C9	120.7 (4)		

### Hydrogen-bond geometry (Å, º)

**Table:**

*D*—H···*A*	*D*—H	H···*A*	*D*···*A*	*D*—H···*A*
N1—H1*A*···O1^i^	0.86	2.46	3.162 (6)	140
N1—H1*B*···O2^ii^	0.86	2.23	3.056 (6)	162
N5—H5*A*···O3^iii^	0.86	2.52	3.285 (6)	148
N5—H5*B*···O4^iv^	0.86	2.20	3.016 (6)	158
C5—H5*C*···O2^v^	0.93	2.56	3.377 (5)	147
C12—H12*A*···N8^iii^	0.93	2.56	3.470 (6)	166

Symmetry codes: (i) −*x*+1, −*y*, *z*+1/2; (ii) −*x*+1/2, *y*, *z*+1/2; (iii) −*x*−1/2, *y*, *z*+1/2; (iv) −*x*, −*y*+1, *z*+1/2; (v) *x*+1/2, −*y*, *z*.
